# OSIRIS-Nose: Organ Sparing Using Interventional Radiotherapy (Brachytherapy) for Invasive Squamous Cell Cancer of the Nasal Vestibule

**DOI:** 10.3390/cancers18050883

**Published:** 2026-03-09

**Authors:** Tamer Soror, Pierre-Alexander Justenhoven, Warren Bacorro, György Kovács, Dirk Rades, Karl-Ludwig Bruchhage, Anke Leichtle

**Affiliations:** 1 Radiation Oncology Department, University of Lübeck/UKSH-CL, 23562 Lübeck, Germany; pierre-alexander.justenhoven@uksh.de (P.-A.J.); dirk.rades@uksh.de (D.R.); 2 Faculty of Medicine and Surgery, University of Santo Tomas, Manila 1015, Philippines; wrbacorro@ust.edu.ph; 3 Department of Radiation Oncology, Benavides Cancer Institute, University of Santo Tomas Hospital, Manila 1015, Philippines; 4 Gemelli-INTERACTS, Università Cattolica del Sacro Cuore, 00168 Rome, Italy; kovacsluebeck@gmail.com; 5 Department of Oto-Rhino-Laryngology and Head and Neck Surgery, University of Lübeck, 23562 Lübeck, Germany; karl-ludwig.bruchhage@uksh.de (K.-L.B.); anke.leichtle@uksh.de (A.L.)

**Keywords:** nose squamous cell carcinoma, nasal vestibule, HDR-brachytherapy, organ preservation, interventional radiotherapy

## Abstract

**Background/Objectives**: Squamous cell carcinoma of the nasal vestibule (SCCNV) represents a rare malignancy traditionally managed by radical surgical resection, frequently at the cost of substantial functional impairment and disfiguring aesthetic consequences. This study investigates an organ-preserving therapeutic strategy integrating high-dose-rate interventional radiotherapy (HDR-IRT; brachytherapy) with organ-preserving surgery. **Material and Methods**: A retrospective analysis of patients with primary SCCNV treated using HDR-IRT between 2008 and 2022, excluding recurrent disease and cutaneous squamous cell carcinomas. Interstitial HDR-IRT catheters were implanted intraoperatively, with radiation delivered twice daily to a target volume encompassing the tumor and a 10–15 mm safety margin. **Results**: Fifty-one patients were included, with a median age of 71 years. The median total dose was 40 Gy. Gross total resection was performed in 7 patients, and subtotal resection in 44. The median follow-up was 35 months. The 5-year nose preservation rate was 90%, with local control at 84%, regional failure-free survival at 94%, and overall survival at 82%. In total, 49 acute toxicity events were documented, including two grade 3 events, while 35 chronic toxicity events were reported, including one grade 3 event. At 3 years, 84.3% of cosmetic outcomes were rated as satisfactory, 9.8% as acceptable, and 5.9% as unsatisfactory. **Conclusions**: The OSIRIS approach, combining HDR-IRT with organ-preserving surgery, is an effective treatment for SCCNV, offering high organ preservation and favorable long-term disease control, with manageable toxicity and positive cosmetic outcomes.

## Simple Summary

Squamous cell carcinoma of the nasal vestibule is a rare tumor commonly treated with radical surgery that may result in permanent functional impairment and facial disfigurement. Developing effective organ-preserving treatment strategies is therefore of major clinical importance. This study evaluates a combined approach integrating conservative surgery with high-dose-rate brachytherapy (HDR-IRT), enabling precise radiation delivery directly to the tumor region. In a cohort of 51 patients, this strategy achieved high rates of local tumor control and long-term nose preservation, while maintaining acceptable toxicity and favorable cosmetic outcomes. These results support HDR-IRT-based organ-preserving treatment as a clinically effective alternative to radical surgery, with the potential to improve both oncologic outcomes and patient quality of life in this rare disease.

## 1. Introduction

Squamous cell carcinoma of the nasal vestibule (SCCNV) represents an exceptionally uncommon and often underrecognized malignancy within the spectrum of head and neck cancers. It constitutes less than 1% of all neoplastic diseases arising in this anatomical region, with a reported annual incidence of approximately 0.41 cases per 100,000 inhabitants [1,2]. Despite its rarity, the clinical and therapeutic implications of SCCNV are significant, owing to the functional and aesthetic importance of the structures it involves. The nasal vestibule itself is a highly specialized anatomical subunit, forming the transitional zone between the external nose and the nasal cavity proper. This region extends from the nostrils posteriorly to the limen nasi and is anatomically delineated by several critical structures, including the columella, the nasal septum, and the medial and lateral crura of the lower lateral cartilages, with some authors suggesting inclusion of the area under the upper lateral cartilage [1,3]. Owing to its unique anatomy, mixed epithelial lining, and prominent aesthetic and functional significance, malignancies of the nasal vestibule pose distinctive diagnostic, therapeutic, and reconstructive challenges, setting SCCNV apart from other tumors of the head and neck [2,3,4]. The location of the nasal vestibule, nestled between the bony nasal cavity and the facial skin, complicates the classification and diagnosis of SCCNV, leading to a lack of consensus on both its definition and staging systems. This ambiguity makes it difficult to assess the prevalence of the condition and complicates the development of universal guidelines for diagnosis and treatment [4]. Recently, the term nasal vestibule has been increasingly used to refer to the area of the nose anterior to the bony piriform aperture, encompassing the entire cartilaginous framework and the underlying area [5,6,7,8,9].

Several staging systems have been proposed for SCCNV, including the Wang system, the American Joint Committee on Cancer (AJCC) staging for non-melanomatous skin cancers (AJCC-NMSC), and the AJCC system for nasal cavity and ethmoid sinus cancers (AJCC-NC) [10,11]. Additionally, a new T classification system is currently under validation in an international multicenter consortium [12,13].

Treatment for SCCNV typically involves either radical surgery or radiation therapy. Many centers regard radical surgery as the standard treatment; however, this often leads to significant functional and aesthetic complications and partial or subtotal loss of the nose [14,15,16]. Radiation treatment has been investigated as an alternative treatment with fewer functional and aesthetic complications [17,18,19,20]. Both external beam radiotherapy (EBRT) and high-dose-rate interventional radiotherapy (HDR-IRT, brachytherapy) have been used to treat SSCNV [21,22]. Regarding EBRT, treatment-related toxicities are frequent, especially in the presence of risk factors [19,20]. Recently, HDR-IRT has been increasingly preferred for its ability to deliver highly conformal radiation in an extreme hypofractionated schedule, resulting in high control rates, reduced damage to surrounding tissues, improved cosmetic results, and high patient satisfaction rates [23,24,25,26,27,28,29].

However, a standardized treatment protocol remains absent, and the choice between these therapies is often determined by tumor stage, location, and the clinical preferences of the treating team. The current analysis examines an alternative treatment strategy that combines HDR-IRT with organ-preserving surgery.

## 2. Materials and Methods

### 2.1. Patients

Following formal approval by the institutional ethics committee, a comprehensive retrospective analysis was undertaken. This review encompassed the medical records of all patients diagnosed with SCCNV who underwent HDR-IRT over a period spanning from January 2008 through December 2022. The study systematically evaluated clinical data, treatment parameters, and documented outcomes to ensure a thorough assessment of this therapeutic approach within the specified timeframe. Patients with recurrent disease and cutaneous squamous cell carcinomas were excluded. The included patients underwent clinical evaluation through physical examination and pan-endoscopy, when needed, for anatomical mapping of the lesion and to exclude deeper extensions and/or other lesions. Complementary contrast-enhanced MRI aided in the local evaluation of the lesion. Neck nodes were evaluated by ultrasound, CT scan, or MRI as needed. The pre-treatment staging was performed according to the AJCC-NC TNM-staging.

A multidisciplinary tumor board specialized in head and neck cancers discussed each patient individually and made treatment recommendations. The tumor board included head and neck surgeons, maxillofacial surgeons, radiation oncologists specialized in interventional radiotherapy, pathologists, radiologists, and nuclear medicine specialists.

### 2.2. Interventional Radiotherapy (Brachytherapy)

Organ-sparing surgery aimed to preserve the structure, function, and aesthetic configuration of the nose. Surgical procedures included gross total resection (GTR), where the entire macroscopic tumor was removed with minimal margins, or subtotal resection (STR), which involved tumor debulking to reduce viable tumor mass. STR was associated with microscopic (R1) or macroscopic residual (R2).

During the same procedure, interstitial HDR-IRT catheters were implanted. The implantation volume encompassed the original macroscopic tumor volume plus a 15–20 mm safety margin. Catheters were inserted transversely or longitudinally to avoid injury to the cartilage of the nasal septum whenever possible. Catheters maintained a parallel relationship with an inter-catheter spacing of 8–12 mm. When needed, an additional intracavitary catheter engulfed within a silicon mold (a single column of Freiburg-flap^®^; Elekta AB, Veenendaal, The Netherlands) was inserted through the nostril.

**Figure 1:**
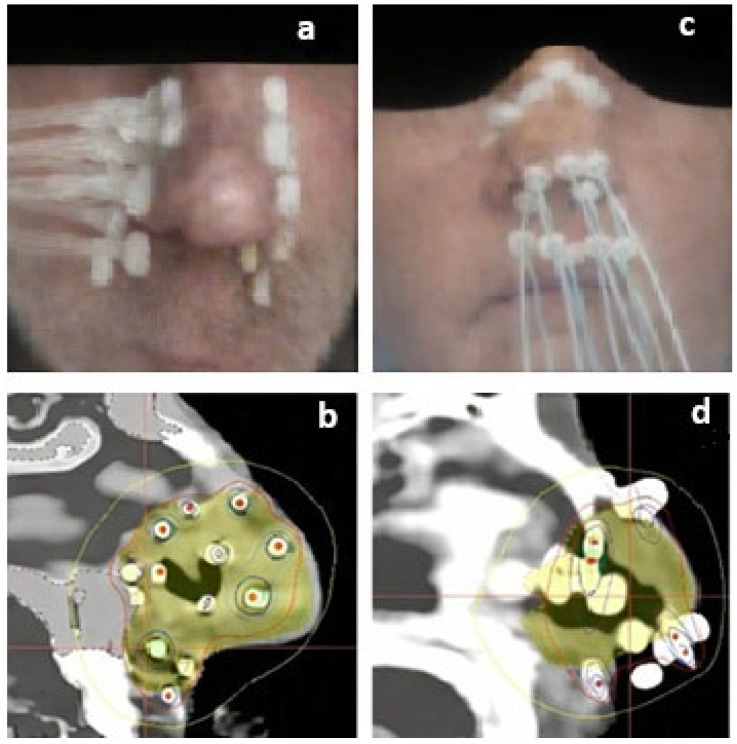
Interstitial HDR-IRT catheters implanted with a representative sagittal CT image showing the dose distribution; (**a**,**b**): transversely, (**c**,**d**): longitudinally. The clinical target volume (CTV) is displayed in yellow, and the 100% prescription isodose line is represented by the red contour.

The following day, a thin-slice simulation CT was performed with radiopaque markers inserted into the catheters. The clinical target volume (CTV) included the estimated original tumor volume plus a 10–15 mm safety margin. The CTV margins were cropped to exclude adjacent bony structures. Dose-volume histogram (DVH) analysis aimed for a coverage index value with 100% of the prescribed dose covering >90% of the target volume, with a dose non-homogeneity ratio (DNR) optimally below 0.35. The maximum dose to bony structures was kept below the prescription dose. Dwell positions were planned across the catheters with 4 mm spacing, ensuring that no dwell positions were placed directly on the skin, Figure 1.

The treatment dose was prescribed based on the target volume, followed by manual dwell time editing and/or graphical optimization as necessary. Beginning in 2019, the inverse planning functionalities available within Oncentra^®^ Brachy (version 4.6, Elekta Brachytherapy Solutions, Veenendaal, The Netherlands) were fully implemented in routine clinical practice to enhance plan quality and reproducibility. Specifically, Inverse Planning Simulated Annealing (IPSA) and Hybrid Inverse Treatment Planning Optimization (HIPO) were employed to automatically generate treatment plans based on predefined dosimetric objectives and organ-at-risk constraints. When necessary, graphical optimization was applied to further refine the dose distribution and adjust dose–volume histogram (DVH) parameters to meet institutional planning criteria. This step allowed individualized fine-tuning in cases where automated optimization alone did not fully satisfy predefined constraints. The prescribed radiation dose was delivered in a twice-daily fractionation schedule, with a minimum interval of six hours between consecutive fractions, in accordance with institutional protocol to allow for normal tissue recovery while maintaining radiobiological effectiveness.

### 2.3. Follow-Up

During the first three years, follow-up evaluations were scheduled at three-month intervals. Subsequently, they were conducted at six-month intervals in the absence of disease recurrence or clinically significant findings. Endoscopic examinations and/or radiological imaging studies were performed as clinically indicated to assess treatment response, verify local control, and investigate suspicious findings detected during routine clinical assessment. Treatment-related toxicities were documented and graded in accordance with the Common Terminology Criteria for Adverse Events (CTCAE) version 5.0. Patient-reported cosmetic outcome was reported by patients on a 3-point scale; satisfactory, acceptable, and unsatisfactory.

### 2.4. Statistical Analysis

The results were presented both as absolute values and as medians, along with their corresponding interquartile ranges. Probability estimates for the local control rate (LCR), Nose preservation rate (NPR), regional nodal control rate (RNCR), distant failure-free survival (DFFS), disease-free survival (DFS), and overall survival (OS) were calculated using the Kaplan–Meier analysis method. As an exploratory analysis, T-stage, surgery, and HDR-IRT dose were analyzed for their impact on local control using Kaplan–Meier analysis and Cox regression, followed by multivariable analysis. Statistical analyses were conducted using SPSS Version 22.0 (IBM Corp., New York, NY, USA) and Jamovi (Version 2.3) (The jamovi project, 2022). Retrieved from https://www.jamovi.org (accessed on 1 March 2026).

## 3. Results

### 3.1. Patients and Tumor Characteristics

A cohort of fifty-one patients underwent organ-preserving surgery combined with and HDR-IRT for SSCNV. Key patient and tumor characteristics are detailed in Table 1. The median age at the time of treatment was 71 years (interquartile range [IQR]: 62–82). Tumor staging revealed a predominance of early disease: 30 patients (59%) were classified as T1, 16 (31%) as T2, 5 (10%) as T3, and 2 (4%) as T4. Remarkably, all patients presented with negative cervical lymph nodes. Five patients underwent unilateral neck dissection due to large tumor or suspected lymphnodes on imaging.

### 3.2. Surgery and Interventional Radiotherapy

GTR was accomplished in 7 patients (13.7%), whereas STR was performed in the remaining 44 patients (86.3%). The median number of catheters implanted was 6 (IQR: 6–9). Patients received a median total HDR-IRT dose of 40 Gy (IQR: 36–45), delivered in a median of 10 fractions (IQR: 9–12) at a median fraction dose of 4 Gy (IQR: 4–5). The median value for coverage index was 90% (IQR: 87.3–90.6), the median DNR value was 0.347 (IQR: 0.323–0.414). Dose parameters of the HDR-IRT are summarized in Table 2.

### 3.3. Treatment Outcome

**Figure 2:**
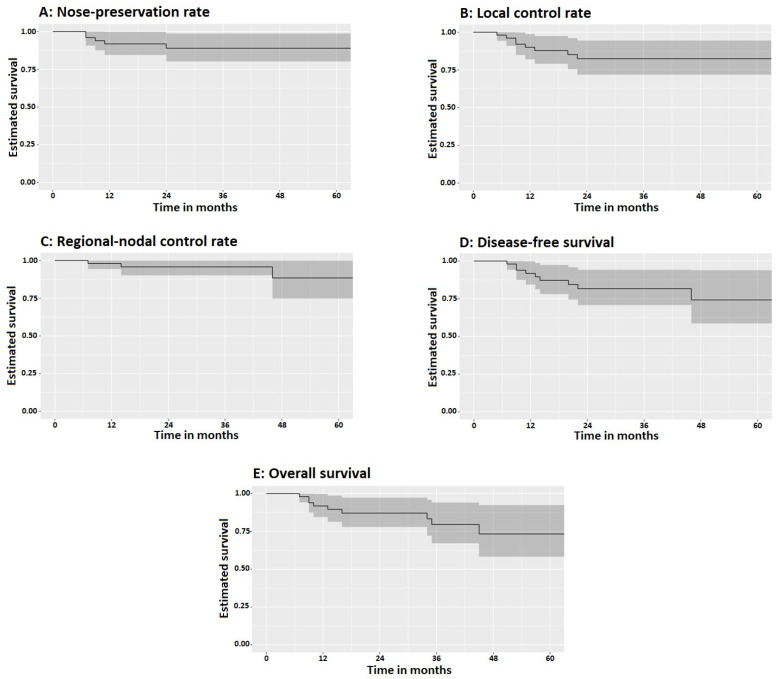
Kaplan–Meier curves showing the probability of different clinical outcomes over time: (**A**) nose-preservation rate, (**B**) local control rate, (**C**) regional-nodal control rate, (**D**) disease-free survival, and (**E**) overall survival. Shaded areas represent the 95% confidence intervals.

The median follow-up period was 35 months (IQR: 20–46). At five years, the nose-preservation rate reached 90% (95% CI: 79–97), with local tumor control achieved in 84% of patients (95% CI: 71–93) and regional nodal control maintained in 94% (95% CI: 84–99). No distant metastases were recorded, with 100% distant failure-free survival at five years. DFS at 5 years was 74% (95% CI: 59–91), while 5-y OS was 82% (95% CI: 69–92), Figure 2. Median survival rate was not reached. All local and regional failures occurred within three years from treatment completion. All deaths occurred in those who developed recurrences.

During follow-up, local recurrences occurred in eight patients; all of them were treated with curative intent. Treatment strategies varied: three patients were re-treated using the same OSIRIS approach, with a reduced prescribed dose of 30 Gy in 10 fractions, and showed no further evidence of disease. The remaining five patients underwent partial or subtotal rhinectomy.

Exploratory statistical analyses were conducted to uncover potential predictors of treatment failure; however, no factors reached statistical significance. Variables examined included T-stage, variations in radiation dose, and the extent of surgical resection (GTR vs. STR). This is likely due to the low number of failure events (8 local, 3 regional, and no distant) and the limited range of doses used (IQR 36–45 Gy, with 86% having received ≥36 Gy; 69%, ≥40 Gy; 29%, ≥45 Gy).

### 3.4. Treatment-Related Toxicities

A total of 49 acute toxicity events were observed, including two grade-3 occurrences and no grade-4 events. Radiodermatitis was the most frequently reported acute toxicity, affecting 57% of patients. Chronic toxicities totaled 35 events, with only a single grade-3 case and no grade-4 events documented (Table 3).

At 3 years, a total of 45 patients were evaluated for cosmetic outcomes. The majority (84.3%) reported being satisfied with their cosmetic outcomes, 9.8% rated the results as acceptable, and only 5.9% considered them unsatisfactory.

## 4. Discussion

The present study investigates an innovative, organ-preserving approach that combines HDR-IRT with minimally invasive surgery for SCCNV. While radical surgery has historically been the cornerstone of SCCNV management due to its definitive local control, its extent often results in significant functional and aesthetic morbidity, including nasal deformation, compromised breathing, chronic pain, and psychological distress [1,2,14,15,16,30,31]. Moreover, the extensive loss of the nose can only be restored through an epithesis or a complex three-stage partial or total nasal reconstruction, requiring three or more surgeries, sometimes up to five years after the first nasal resection, which could imply an immense burden on patients and their families [32,33,34].

The current study integrates organ-sparing surgery with HDR-IRT, where the surgical aim is to debulk the viable tumor, thereby reducing tumor volume. This reduction plays a critical role in enhancing local control rates when brachytherapy is used as the sole treatment for nasal cancers [35].

The lack of standardized staging for SCCNV remains a challenge, complicating comparisons across studies and the development of universally accepted treatment guidelines. Several staging systems, such as the Wang system, AJCC-NMSC, and AJCC-NC, currently coexist, contributing to heterogeneity in patient management [11,12]. In the current study, the AJCC-NC staging system was used, explaining the inclusion of five patients with T3–4 disease who were technically feasible for the OSIRIS approach. Furthermore, harmonizing staging systems through international consensus would significantly facilitate comparative research, allowing more robust conclusions regarding the most effective therapeutic strategies [12,13].

Recently, the New Rome classification for squamous cell carcinoma of the nasal vestibule (SCCNV) was validated in a large multicentric international study encompassing over 600 patients. This study demonstrated that the New Rome classification offers superior predictive and descriptive accuracy compared to the previously established staging systems, including the Wang, AJCC-NMSC, and AJCC-NC classifications [13]. By integrating factors such as tumor depth, anatomical subsite involvement, and local invasiveness, the New Rome system provides a more nuanced stratification that can better guide treatment planning and prognostication.

Applying the New Rome classification to the T3 and T4 patients within our cohort results in a downstaging to T2. This finding highlights the potential for the New Rome system to refine risk assessment and may have important clinical implications as patients previously considered high-stage may, in fact, have tumors with more favorable biological behavior than initially assumed. Such reclassification could influence decisions regarding the aggressiveness of surgery, the intensity of adjuvant therapy, and follow-up strategies. Moreover, it underscores the evolving understanding of SCCNV as a distinct clinical entity, for which traditional head and neck cancer staging systems may not fully capture the prognostic nuances.

The OSIRIS approach utilized modern techniques of brachytherapy, including anatomical implantation, image-guided planning using thin-slice CT, inverse treatment planning, and personalized dose optimization. Moreover, the prescribed dose and fractionation ensured a short overall treatment duration, maximizing the biological effectiveness of radiation in tumor cell eradication [36]. This study achieved optimal dose coverage, with median coverage indices around 90%, and minimal dose heterogeneity reflected by a favorable median DNR (˂0.35), aligning with the recent international recommendations [37].

The dose and fractionation schedule used in the current study are consistent with the most recent recommendations of GEC-ESTRO. These guidelines recommend 45–54 Gy delivered in 3 Gy fractions, 40–44 Gy delivered in 4 Gy fractions, or a total dose of 44 Gy in a mixed schedule consisting of 4 Gy for the first and last fractions with 12 intermediate fractions of 3 Gy. In the present study, the median total dose was 40 Gy, with a median fraction size of 4 Gy [37].

A systematic review by Tagliaferri et al. investigated the role of interventional radiotherapy for squamous cell carcinoma of the nasal vestibule and reported that five-year local control rates ranged from 69% to 97% [38]. In the present study, we observed high local control (84% at 5 years) and nose-preservation rates (90% at 5 years), which compare favorably with prior studies and the findings of the systematic review [23,24,25,26,27,28,35,38]

Additionally, an extensive Dutch multicenter analysis examined the use of radiation therapy as a nose-preservation strategy for nasal vestibule cancers. This study compared brachytherapy with EBRT and found that brachytherapy provided significantly higher 3-year local control (95% vs. 71%, *p* < 0.01) and superior nasal preservation rates (82% vs. 61%, *p* < 0.01). However, no difference was observed in survival outcomes between the two treatment modalities [39].

One notable strength of the OSIRIS protocol is the achievement of excellent regional nodal control (94%) and the absence of distant metastases. This finding supports brachytherapy alone as upfront management, and withholding nodal irradiation in N0 disease. These results are consistent with previously published data, which emphasize the predominantly localized nature of SCCNV, thereby validating the importance of effective local therapy in controlling disease progression [12,15,19]. The absence of distant metastases underscores the localized progression of SCCNV, further advocating for intensive local management strategies such as HDR-IRT.

Exploratory statistical analysis performed within this study aimed to identify factors predictive of treatment failure, but did not yield significant correlations, despite analyzing variables such as T-stage, surgical strategy, and radiation dosage. This could be explained by the relatively few failure events and the small range of radiation doses prescribed. However, one study used image-guided brachytherapy and found that a larger tumor volume (≥2.3 cc) resulted in worse 3-year regional control compared to smaller tumors [36]. The lack of significant multivariable predictors in our study is likely attributable to the limited sample size and retrospective design, which inherently carries risks of selection bias and incomplete data reporting. Nevertheless, the reported evidence strongly supports volume-based risk stratification in treatment planning and implementing adaptive dosing or elective nodal strategies for larger tumors.

A pivotal outcome of this study is the favorable toxicity profile associated with the OSIRIS approach. Acute toxicities were predominantly mild, with grade 1–2 radiodermatitis, and only a single case of grade 3 acute dermatitis was recorded. Chronic toxicities were similarly limited, predominantly involving manageable conditions such as telangiectasia, peripheral sensory neuropathy, anosmia, and depigmentation. Only one grade-3 chronic event was documented. This favorable toxicity profile compares favorably with both radical surgery, which carries higher morbidity, and external beam radiotherapy, which traditionally presents a broader irradiation field and a potential for increased damage to adjacent normal tissues [17,18,26].

The Dutch multicenter analysis reported contradictory toxicity results. Despite the superior local control and nasal preservation achieved with brachytherapy over EBRT, grade ≥2 toxicity was higher following brachytherapy (20% vs. 3%, *p* = 0.03), with radiation ulcers being the most common complication [39]. In the current study, only three patients developed ulcers. This lower rate may be attributed to a meticulous review of DVH parameters and ensuring that no dwell positions were placed directly on the skin.

Our study showed a very high rate of patient-reported satisfaction with cosmetic outcomes. These findings align with previous studies on HDR-IRT, many of which reported similarly high to very high levels of patient satisfaction with cosmetic results [25,26,35]. Aesthetic outcomes typically depend on the size and location of the primary lesion. After treatment, tissue defects or nasal deviations resulting from fibrosis can significantly impact the cosmetic result. To minimize recall bias, patient satisfaction should ideally be assessed both before and after treatment, establishing baseline measurements for comparison.

Despite the encouraging outcomes presented, several limitations must be acknowledged. Firstly, the study’s retrospective design limits the strength of causal inferences. Additionally, the sample size, though consistent with the rarity of SCCNV, restricts statistical power and generalizability. Prospective multicentric trials with more extensive patient cohorts are needed to confirm these promising results, to generalize the findings, and to establish interventional radiotherapy as a standard care strategy potentially.

## 5. Conclusions

The OSIRIS approach, combining HDR-IRT with organ-preserving surgery, appears to be a promising treatment strategy for SCCNV. It is associated with high rates of organ preservation, favorable long-term disease control, acceptable treatment-related toxicity, and positive patient-reported cosmetic outcomes. These findings support further investigation of this approach as a potential alternative to radical surgery in the management of SCCNV. These promising results support the broader adoption and further evaluation of combined HDR-IRT and organ-preserving surgical approaches. To validate these findings and refine clinical management guidelines for SCCNV, future prospective, multicenter studies and comparative analyses are strongly needed.

## Figures and Tables

**Table 1:** Patients and tumor characteristics (n = 51).

Sex		
Male–female ratio	1.32	
	Median	IQR
Age	71	(62–82)
Diameter (in cm)	1.9	(1.15–2.6)
		
	n	(%)
T Stage		
T1	30	59
T2	16	31
T3	3	6
T4	2	4
N Stage		
cN0	46	90
pN0	5	10
Grade		
G1	11	21.5
G2	18	35.3
G3	16	31.4
Gx	6	11.8
Margin status		
Negative margin (R0)	6	11.8
Positive margins (R1/R2)	45	88.2
Lymph-vascular invasion	1	2%
Perineural invasion	2	4%

IQR, interquartile range.

**Table 2:** Dose parameters of the HDR-IRT.

	Median	IQR
Dose and Fractionation		
Dose (in Gray)	40	(36–45)
Fraction size (in Gray)	4	(4–5)
Number of fractions	10	(9–12)
Overall treatment time (in days)	7	(6–8)
Coverage parameters		
Coverage index (V100)	90%	87.3–90.6
DNR (V150/V100)	0.347	(0.323–0.414)
D90	100.1%	(96.1–100.8)
V150	37.7%	(31.9–46.9)
V200	15.1%	(12–21.9)
Target minimum dose (D100)	60%	(57–68.3)

IQR, interquartile range.

**Table 3:** Acute and late toxicities.

Acute	*N	(%)
Grade 1–2		
Dermatitis	28	57
Edema	8	22
Epistaxis	3	10
Mucositis	2	6
Grade 3		
Dermatitis	2	4
**Late**		
Grade 1–2		
Telangiectasia	8	23
Peripheral sensory neuropathy (allodynia or dysesthesia)	6	17
Anosmia	5	14
Depigmentation	5	14
Pain	4	11
Dysgeusia	2	6
Fibrosis	2	6
Chronic ulcer	2	6
Grade 3		
chronic ulcer	1	3

*N, number.

## Data Availability

Further data could be submitted on request according the institutional rules of patient’s data management.

## Abbreviations

AJCC	American Joint Committee on Cancer
AJCC-NC	AJCC system for nasal cavity and ethmoid sinus cancers
AJCC-NMSC	AJCC system for non-melanomatous skin cancers
CI	Confidence interval
CT	Computed tomography
CTCAE	Common Terminology Criteria for Adverse Events
CTV	Clinical target volume
DFFS	distant failure-free survival
DFS	Disease free survival
DNR	Dose non-homogeneity ratio
DVH	Dose-volume histogram
EBRT	External beam radiotherapy
GTR	Gross total resection
Gy	Gray
HDR-IRT	High-dose-rate interventional radiotherapy
HIPO	Hybrid Inverse Treatment Planning Optimization
IPSA	Inverse Planning Simulated Annealing
IQR	interquartile range
MRI	Magnetic resonance imaging
NPR	Nose preservation rate
OS	Overall survival
OSIRIS	Organ Sparing using Interventional Radiotherapy (Brachy-therapy) for Invasive Squamous cell cancer of the Nasal vestibule
RNCR	regional nodal control rate
SSCNV	Squamous cell carcinoma of the nasal vestibule
STR	Subtotal resection
